# End-stage renal disease and low level exposure to lead, cadmium and mercury; a population-based, prospective nested case-referent study in Sweden

**DOI:** 10.1186/1476-069X-12-9

**Published:** 2013-01-23

**Authors:** Johan Nilsson Sommar, Maria K Svensson, Bodil M Björ, Sölve I Elmståhl, Göran Hallmans, Thomas Lundh, Staffan MI Schön, Staffan Skerfving, Ingvar A Bergdahl

**Affiliations:** 1 Department of Public Health and Clinical Medicine, Occupational and Environmental Medicine, Umea University, Umea, Sweden; 2 Department of Molecular and Clinical Medicine-Nephrology, Institute of Medicine, Sahlgrenska Academy at University of Gothenburg, Gothenburg, Sweden; 3 Department of Health Sciences, Division of Geriatric Medicine, Lund University, Lund, Sweden; 4 Department of Public Health and Clinical Medicine, Nutritional Research, Umea University, Umea, Sweden; 5 Division of Occupational and Environmental Medicine, University Hospital, Lund, Sweden; 6 Diaverum Renal Services Group, Lund, Sweden & Swedish Renal Registry, Jönköping, Sweden

**Keywords:** Biobank, Cadmium, Case-referent, End-stage renal disease, Lead, Mercury

## Abstract

**Background:**

Cadmium (Cd), lead (Pb), and mercury (Hg) cause toxicological renal effects, but the clinical relevance at low-level exposures in general populations is unclear. The objective of this study is to assess the risk of developing end-stage renal disease in relation to Cd, Pb, and Hg exposure.

**Methods:**

A total of 118 cases who later in life developed end-stage renal disease, and 378 matched (sex, age, area, and time of blood sampling) referents were identified among participants in two population-based prospective cohorts (130,000 individuals). Cd, Pb, and Hg concentrations were determined in prospectively collected samples.

**Results:**

Erythrocyte lead was associated with an increased risk of developing end-stage renal disease (mean in cases 76 μg/L; odds ratio (OR) 1.54 for an interquartile range increase, 95% confidence interval (CI) 1.18-2.00), while erythrocyte mercury was negatively associated (2.4 μg/L; OR 0.75 for an interquartile range increase, CI 0.56-0.99). For erythrocyte cadmium, the OR of developing end-stage renal disease was 1.15 for an interquartile range increase (CI 0.99-1.34; mean Ery-Cd among cases: 1.3 μg/L). The associations for erythrocyte lead and erythrocyte mercury, but not for erythrocyte cadmium, remained after adjusting for the other two metals, smoking, BMI, diabetes, and hypertension. Gender-specific analyses showed that men carried almost all of the erythrocyte lead and erythrocyte cadmium associated risks.

**Conclusions:**

Erythrocyte lead is associated with end-stage renal disease but further studies are needed to evaluate causality. Gender-specific analyses suggest potential differences in susceptibility or in exposure biomarker reliability.

## Background

Chronic kidney disease and development of end-stage renal disease is of increasing public health concern worldwide. In Sweden, the number of patients treated for end-stage renal disease is increasing, and diabetes and nephrosclerosis due to hypertension are the most common causes of end-stage renal disease [1]. A higher prevalence of obesity, resulting in an increase of diabetes and hypertension contribute [2]. Obesity, *per se*, is also associated with the development of chronic kidney disease in both diabetic and non-diabetic renal disease [3-5]. Other risk factors for chronic kidney disease in the general population are old age, presence of any degree of chronic kidney disease (estimated glomerular filtration rate), proteinuria, smoking, and hypertension [6]. In patients with chronic kidney disease, low renal function (estimated glomerular filtration rate) and severe albuminuria independently predict end-stage renal disease [7]. Possibly, environmental exposure to nephrotoxicants, especially in susceptible individuals, (i.e., patients with diabetes, hypertension, and preexisting chronic kidney disease), may contribute to the disease and/or its progression. The metals cadmium (Cd; [8]), mercury (Hg; [9]) and lead (Pb; [10,11]) at high exposures in occupational settings are known to be nephrotoxic.

Renal effects of Cd (e.g. [12]) and Pb (e.g. [13,14]) have been suggested even at low-level exposures in the general population such as increased levels of urinary beta-microglobulin, increases in blood urea nitrogen, serum creatinine, creatinine clearance (estimated creatinine clearance), or glomerular filtration rate (estimated glomerular filtration rate). However, the clinical relevance of these biomarkers can be questioned and has been challenged.

A clinically relevant hard renal endpoint is the development of end-stage renal disease, requiring renal replacement therapy (i.e., dialysis or transplantation). An ecological study indicated that the risk of end-stage renal disease increased by proximity to Cd-emitting industries [15], and cross-sectional studies indicate that patients with chronic kidney disease have increased concentrations of Cd and Pb as compared to referents [16-20], and that Pb is related to progression of chronic kidney disease [21,22]. However, these findings may be a result of reverse causation, i.e., the disease itself, or its treatment, affects metal retention. In addition, high exposure to inorganic Hg is related to development of membranous glomerulonephritis with nephrotic syndrome in susceptible individuals [23].

Up until now there are, to our knowledge, no prospective studies, targeting associations between the development of end-stage renal disease and effects of low level exposure to Cd, Pb, and Hg. The aim of the present study was to elucidate how erythrocyte concentrations of Pb (Ery-Pb), Cd (Ery-Cd), and Hg (Ery-Hg) in the general population relate to the risk of developing end-stage renal disease later in life. This was done in a population-based case-referent study nested within two prospective cohorts from Northern and Southern Sweden with stored blood samples, collected before diagnosis of end-stage renal disease.

## Methods

### Subjects

#### The northern Sweden health and disease study

The Northern Sweden Health and Disease Study [24] includes the population-based Västerbotten Intervention Project [25] and the Northern Sweden WHO Monitoring of Trends and Cardiovascular Disease (MONICA) Study, both of which include anthropometric, blood-pressure, and lifestyle data, as well as the local Mammography Screening Project, with more limited data. The Västerbotten Intervention Project was initiated in 1985 as a health-promotion program for the population in one county (Västerbotten; ca. 250,000 inhabitants). Every year, inhabitants reaching the ages of 40, 50, and 60 (during some years also 30) are invited to their local health centers for a routine health screening. MONICA was also initiated in 1985 as a health examination program for cardiovascular disease and diabetes, where randomly selected inhabitants were invited to participate in a continuous health survey. In addition, women in Västerbotten County aged approximately 40–70 years have been encouraged to take part in a mammographic screening program every two to three years since 1995. Participants in these programs are asked to donate blood samples, which are then stored in a biobank (−70°C). Between the years 1985–2002, a total of 74,000 unique subjects had donated blood samples to the Northern Sweden Health and Disease Study.

The participation rate during 1985–2002 was approximately 60% for the Västerbotten Intervention Project, 77.2% for MONICA, and 65% for the Mammography Screening Project. The collection of samples is still continuing.

#### The Malmö diet and cancer study

The Malmö Diet and Cancer study [26] is a prospective population-based study with anthropometric, blood-pressure, and lifestyle data from a baseline examination between 1991–1996 of 45–64 year-old men and women (n = 30,447) living in the city of Malmö in Southern Sweden. Subjects from the municipal registry were randomly invited to participate from the total source population of 74,138. Recruitment was also made using local advertisements, and by recommendations from participants. The only exclusion criterion for participation was lack of Swedish language skills. Participants who emigrated during the study period were censored on the date they left the country. Donated blood samples were stored in a biobank (−70°C). The participation rate was approximately 45%.

### Follow-up and case-referent study design

Since 1991, the Swedish Renal Registry (SRR; [27]) collects data on all patients with end-stage renal disease (defined as glomerular filtration rate <10-15 ml/min), starting renal replacement therapy, i.e., dialysis or transplantation. All units performing dialysis and/or kidney transplantation in Sweden report to the SRR (attaining data from >95% of all patients).

**Figure 1:**
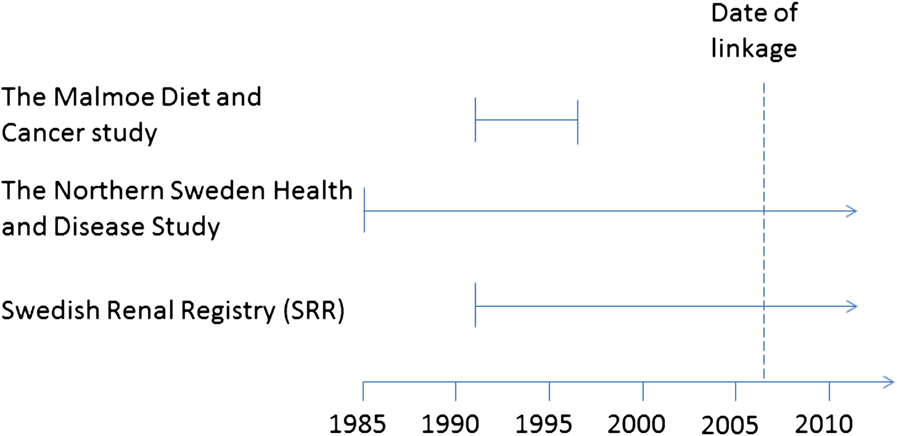
**Time period of recruitment and blood sampling within the Malmö Diet and Cancer study and The Northern Sweden Health and Disease study, from which participants for the present study were selected.** The identities of the participants were linked to the Swedish Renal Registry for identification of end-stage renal disease cases who had donated a blood sample in any of the cohort studies. Vertical lines indicate beginning and end of cohort recruitment, and arrows indicate that recruitment is still continuing.

Subjects who participated in any of the two cohorts, and later (up to 31 December 2006) developed end-stage renal disease, were identified by linkage to the SRR using their unique personal identity number (Figure 1). The case with the oldest blood sample was obtained in 1993.

**Figure 2:**
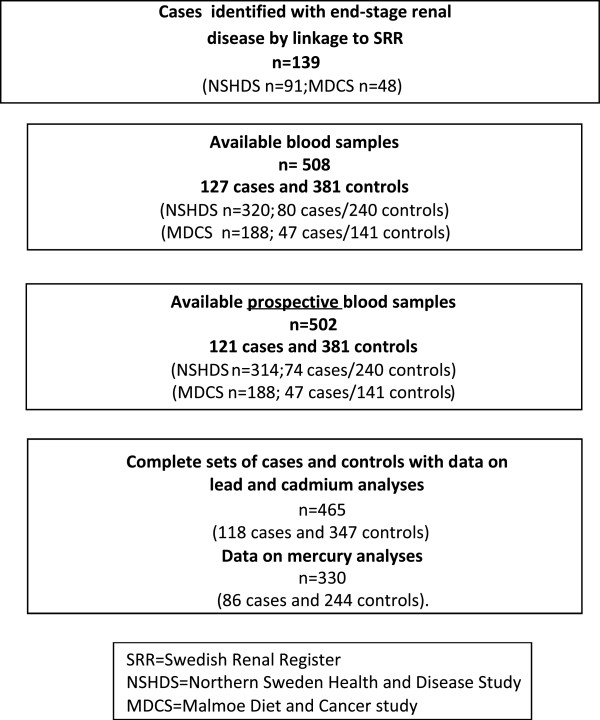
Flowchart of number of individuals.

For each case, three referents without end-stage renal disease, alive at time of the end-stage renal disease diagnosis (as ascertained through linkage to the Swedish National Death Registry), were individually matched for cohort, age, sex, and time of sampling (Figure 2). For the 139 identified cases and 382 referents, Ery-Cd and Ery-Pb were analyzed in samples from 118 cases and 378 referents, and Ery-Hg in 87 cases and 288 referents. Complete sets of cases and referents with Ery-Cd and Ery-Pb analyses were obtained for 118 cases and 347 referents, and 86 cases and 244 referents with Ery-Hg.

Background information on smoking (Mammography Screening Project: only yes/no; other cohorts: current, ex-, never), blood pressure, diabetes, and hypertension (i.e. having systolic blood pressure >140, diastolic >90 mm Hg, or being treated for high blood pressure), was obtained from the baseline health examinations.

All subjects gave informed written consent to their participation in the study, which was approved by the ethical committee of Umeå University.

### The choice of erythrocyte concentrations as biomarker

Metals were analyzed in erythrocytes, and neither urine nor whole-blood samples were available. The most commonly used biomarkers for cadmium are the concentrations in urine and whole blood. Urinary cadmium reflects mainly cadmium in the kidney, in turn reflecting long-term exposure; meanwhile, whole-blood cadmium reflects a combination of both current and historical exposure. Since almost all blood cadmium is present in erythrocytes, cadmium in whole blood and in erythrocytes reflects the same time period of exposure [8]. An advantage with both whole blood and erythrocyte cadmium, compared with urinary cadmium, is that the blood markers are not affected by tubular function. However, a disadvantage of using Ery-Cd is that it not only reflects long-term accumulation of cadmium body burden that may be more relevant for kidney effects.

For lead, the most commonly used exposure indicator is the concentration in whole blood. Most of the lead in whole blood is bound to proteins in the erythrocytes [8]; the lead concentration in erythrocytes is approximately two times the lead concentration in whole blood.

Ery-Hg reflects both inorganic mercury and methylmercury exposure, though a stronger influence from the latter is to be expected. Over 90% of the methylmercury in whole blood is in erythrocytes, while inorganic (mercuric) mercury distributes almost equally between plasma and erythrocytes, with perhaps somewhat more in plasma [9].

### Chemical analyses

Ery-Cd and Ery-Pb were determined by inductively coupled plasma-mass spectrometry (ICP-MS; Thermo X7, Thermo Elemental, Winsford, UK; [28]).

The detection limits, calculated as 3 times the standard deviation of the blank, were 0.04 and 0.11 μg/L, respectively. Analytical accuracy was checked against reference materials. For Seronorm Trace elements whole blood (Seronorm; Lot MR4206, 503109, SERO AS, Billingstad, Norway), the results obtained for Cd were 0.67 ± 0.04 (mean ± standard deviation (SD); n = 21) and 5.6 ± 0.18 (n = 21) μg/L, respectively (recommended 0.68–0.80 and 5.6–6.4 μg/L), and for Pb 26.2 ± 0.53 (n = 21) and 372 ± 12 (n = 21) μg/L, respectively (recommended 26.2–29.0 and 372–414 μg/L). For the human blood reference samples from the Centre de Toxicologie du Quebec, Canada, the obtained values for Cd and Pb were: Cd (Lot C0515) 0.73 ± 0.03 (n = 21), recommended 0.79 ± 0.23; and Pb (Lot L0608) 31.2 ± 0.68 μg/L, recommended 31.1 ± 4.7 μg/L.

Ery-Hg was determined by cold vapor atomic fluorescence spectrophotometry [29]. The detection limit was 0.08 μg/L. The analytical accuracy for Hg in blood (Seronorm; Lot MR4206, 0512627) was 1.96 ± 0.10 (n = 49) and 15.0 ± 0.44 (n = 49) μg/L, respectively (recommended 2.0–2.4 and 16.1–19.7 μg/L). For the samples from the Centre de Toxicologie du Quebec, Canada (Lot M0408) results were 1.94 ± 0.13 μg/L (recommended 2.0 μg/L). Due to limited availability of samples for analyses, Ery-Hg was only determined in those samples that had sufficient remaining sample volume after the detection of Cd and Pb.

All determinations were made in duplicate preparations. The method imprecisions, calculated as the coefficient of variation (CV%), were 5.7%, 1.3%, and 8.6%, for Cd, Pb, and Hg, respectively.

### Statistical analyses

The association between end-stage renal disease and each of the potential risk factors Ery-Cd, Ery-Pb, Ery-Hg, BMI, diabetes, smoking, and hypertension, were calculated using univariate conditional logistic regression based on the matched sets of cases and referents. In addition to the univariate analyses, Spearman’s correlation coefficient, or odds ratio (OR), was calculated between each pair of candidate risk factors to be able to investigate if the candidate risk factors were confounders, changed the effects of the metals, or were independent risk factors. Finally, a multiple conditional logistic regression model was obtained using a backwards stepwise procedure where variables that did not statistically significantly explain variation in end-stage renal disease risk and did not change the effects of Ery-Cd, Ery-Pb, or Ery-Hg more than 10% were excluded.

Univariate analysis was also performed, categorizing Ery-Cd, Ery-Pb, and Ery-Hg using quartile limits which were calculated using concentrations from cases only, to obtain equal number of cases in each category. Quartile categorization as well as penalized cubic splines were used to assess linearity between independent variables and log-odds. Sensitivity analyses were performed by excluding influential observations. In addition, for the purpose of assessing model fits, the Hosmer-Lemeshow goodness-of-fit test was used by including the matching factors as covariates in a logistic regression model. However, all results shown in the tables and figures are based on conditional logistic regression, using matched sets of cases and referents. Time between sampling and end-stage renal disease as a confounder between metals and end-stage renal disease was investigated by estimating the associations between metals and end-stage renal disease in subsets of time between sampling and end-stage renal disease defined by quartile limits.

All statistical analyses were performed using the statistical package R, version 2.11.0 [30]. P-values <0.05 were considered statistically significant.

## Results

Characteristics of the study participants at baseline, i.e., before any of the cases had developed end-stage renal disease, are presented in Table 1. There were differences in the occurrence of diabetes and hypertension between cases and referents, and a tendency towards a difference in smoking habits.

**Table 1:** Baseline data on cases that later in life developed end-stage renal disease and referents

	**Cases**	**Referents**	**Difference between cases and referents with 95% confidence intervals/p-value**
Cases and referents from both study populations	118	347	
Number of men/women	66/52	194/153	
Northern Sweden Health and Disease Study (NSHDS)	72	214	
Malmoe Diet and Cancer Study (MDCS)	46	133	
Characteristics at base-line examination, i.e. prior to diagnosis of end-stage renal disease:			
Diabetes (y/n)	20 (21%)	8 (3%)	OR = 8.3^a^ (3.5, 22)
Blood pressure (mm Hg), mean(standard deviation)			
Systolic (SBP)	141 (21.3)	135 (19.9)	5.8 ^a^ (1.5, 10)
Diastolic (DBP)	85.8 (11.5)	83.1 (9.6)	2.7 ^a^ (0.42, 5.0)
Hypertension^b^ (y/n)	66 (54.6%)	125 (34.8%)	OR = 2.2^a^ (1.4, 3.5)
Smoking			p = 0.091^a^
Never-smoker	34 (31%)	141 (41%)	
Not current smoker (undefined ex-smoker or never-smoker)	11 (10%)	38 (11%)	
Ex-smoker	26 (24%)	79 (23%)	
Current smoker	39 (35%)	82 (24%)	
Mean age at baseline^c^ (years)	63 (40–80)	63 (40–80)	
Mean age at diagnosis of end-stage renal disease (years)	71 (46–87)		
Time from baseline examination to development of end-stage renal disease (years)	7.7 (1–16)		

OR=odds ratio.

^a^Differences in binary variables were expressed as ORs with 95% confidence intervals, continues variables as the difference with 95% confidence intervals, and p-values using the chi-square test were reported for categorical variables with more than two levels.

^b^Hypertension was defined as SBP >140 or DBP > 90 mm Hg, or antihypertensive medication at baseline.

^c^matching factor.

Furthermore, statistically significant differences were found in Ery-Cd (geometric mean among cases 0.86 μg/L and referents 0.66 μg/L, geometric mean 23% lower among referents with 95% CI 8.2-44.7%), Ery-Pb (geometric mean among cases 66.2 μg/L and 55.0 μg/L among referents, geometric mean 17% lower among referents with 95% CI 7.8-29%) and Ery-Hg (arithmetic mean among cases 2.44 μg/L and referents 3.06 μg/L, 0.62 μg/L difference with 95% CI 0.17-1.08 μg/L) between cases and referents. The largest differences were found for cases with nephrosclerosis, glomerulonephritis, and pyelonephritis as the primary cause of end-stage renal disease, while the smallest differences were found for cases with diabetic nephropathy and non-specified diagnoses (See Additional file 1: Table S1).

To investigate if candidate risk factors were confounders, effect modifiers, or independent risk factors, and to identify possible multicollinearity between these variables, the correlation coefficient between each pair of risk factors was calculated (See Additional file 1: Table S2). Ery-Cd and Ery-Pb were statistically significantly correlated (Spearman’s correlation coefficient 0.19, 95% CI 0.10-0.27), and Ery-Pb was near-significantly correlated (Spearman’s correlation coefficient 0.10, 95% CI 0.00-0.20) with Ery-Hg. Both Ery-Cd and Ery-Pb were positively associated with age and smoking.

**Figure 3:**
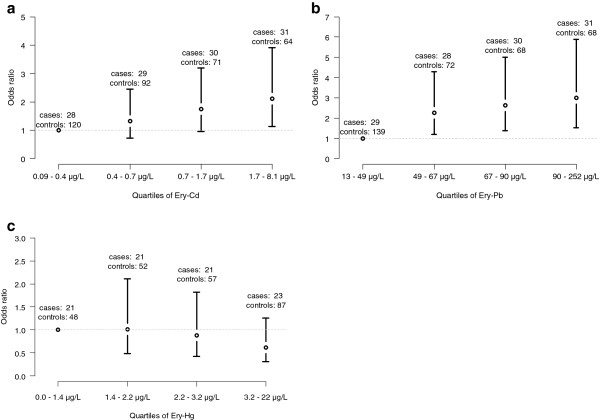
**Univariate association between erythrocyte concentrations of cadmium (Ery-Cd; a), lead (Ery-Pb; b), and mercury (Ery-Hg; c), and risk of developing end-stage renal disease.** Quartile concentration limits were calculated from cases.

Univariate analysis showed that increased Ery-Pb and Ery-Cd were associated with increased risk of end-stage renal disease, however the association was not statistically significant for Ery-Cd (odds ratio (OR) 1.010 per μg/L increase with 95% CI 1.004-1.016, and OR 1.17 per μg/L increase with 95% CI 0.99-1.38 respectively). Ery-Hg was negatively associated with the risk of developing end-stage renal disease: OR 0.86 95% CI 0.74-0.996. Corresponding ORs for an interquartile range increase were 1.15, 1.54, and 0.75 for Ery-Cd, Ery-Pb, and Ery-Hg, respectively (Table 2). To investigate the shape of the associations, analyses were also made categorizing Ery-Cd, Ery-Pb, and Ery-Hg by quartile limits among cases (Figure 3a-c). More elaborate figures justifying model assumptions are shown as supplementary material (See Additional file 1: Figure S1).

**Table 2:** Univarite modeling of the association between end-stage renal disease and candidate risk factors

**Variable**	**Number of individuals**	**OR**	**95% CI**	**IQR**	**OR**_**IQR**_	**95% CI**
Ery-Cd (μg/L)	465	1.17	(0.99, 1.38)	0.37-1.3	1.15	(0.99, 1.34)
Ery-Pb (μg/L)		465	1.010	(1.004, 1.016)	39.8-83.5	1.54	(1.18, 2.00)
Ery-Hg (μg/L)	330	0.86	(0.74, 0.996)	1.53-3.5	0.75	(0.56, 0.99)	
BMI (kg/m^2^)	451	1.05	(0.99, 1.11)	23.5-28.1	1.24	(0.97, 1.60)	
Diabetes (y/n)	381	13.1	(4.46, 38.7)				
Hypertension^a^ (y/n)	344	1.58	(0.90, 2.76)				
Smoking	442						
Never-smoker	172	1					
Not current smoker (undefined ex-smoker or never-smoker)	49	1.29	(0.33, 5.10)				
Ex-smoker	101	1.53	(0.83, 2.82)				
Current smoker	120	2.15	(1.16, 3.98)				

Detailed legend: Univariate modeling of associations between end-stage renal disease and erythrocyte metal concentrations, body mass index (BMI), diabetes, hypertension and smoking at baseline. For continuous variables, ORs are given for both unit increase and for an interquartile range increase.

OR=odds ratio per unit increase. CI=confidence interval. IQR= interquartile range. OR_IQR_=odds ratio for an interquartile range increase. BMI=Body Mass Index.

^a^Hypertension was defined as SBP >140 or DBP > 90 mm Hg, or antihypertensive medication at baseline.

Using multiple regression analysis adjusting for all other statistically significant variables or variables that changed the effect of the metals, both Ery-Pb and Ery-Hg were statistically significantly related to the development of end-stage renal disease (Table 3). The OR for one μg/L increase of Ery-Pb was 1.013 (95% CI 1.003-1.023) and for Ery-Hg 0.81 (95% CI 0.668-0.99), when also taking into consideration diabetes, BMI and hypertension. The modifications of ORs between univariate and multiple conditional logistic regression analysis were due to effect modification by covariates and not because of change in selection of individuals when restricting to those with complete data sets (data not shown). Ery-Cd was not statistically significant in the multiple model (OR = 1.11; 95% CI 0.78-1.57; not in table). The change in estimate for each metal exposure variable by including the two other respective metals is shown as supplementary material (See Additional file 1: Figure S2).

**Table 3:** Multiple modeling of the association between end-stage renal disease and risk factors

**Multiple model**					
Number of individuals = 244	**OR**	**95% CI**	**IQR**	**OR**_**IQR**_	**95% CI**
Ery-Pb (μg/L)	1.013	(1.003, 1.023)	0.37-1.3	1.74	(1.13, 2.68)
Ery-Hg (μg/L)	0.81	(0.66, 0.99)	39.8-83.5	0.66	(0.44, 0.98)
BMI (kg/m^2^)	1.13	(1.02, 1.25)	1.53-3.5	1.75	(1.10, 2.79)
Diabetes (y/n)	35.8	(4.37, 294)			
Hypertension^a^ (y/n)	4.23	(1.97, 9.11)			

Detailed legend: Multiple modeling of the associations between end-stage renal disease and erythrocyte concentrations of lead and mercury, taking into consideration the covariates at baseline that either were statistically significant or changed any of the metal estimates.

OR=odds ratio for a unit increase. CI=confidence interval. IQR= interquartile range. OR_IQR_=odds ratio for an interquartile range increase. BMI=Body Mass Index.

^a^Hypertension was defined as SBP >140 or DBP > 90 mm Hg, or antihypertensive medication at baseline.

Gender-specific analyses taking into consideration all statistically significant variables and variables changing the metal estimates indicated a stronger dose–response relation among men than women for Ery-Cd (men: OR 1.39 (95% CI 0.96-2.00; N = 151); women OR 0.79 (95% CI 0.51-1.23; N = 93)) and Ery-Pb (men: OR 1.014 (95% CI 1.0023-1.026; N = 151); women OR 1.00 (95% CI 0.98-1.022; N = 93)). Analyses also suggested a negative association with Ery-Hg in women not evident among men (men: OR 0.89 (95% CI 0.71-1.13; N = 151); women OR 0.68 (95% CI 0.47-0.98), N = 93).

In addition, a separate analysis was also made for individuals with more than median (8 years) duration between blood sampling and diagnosis of end-stage renal disease, taking into consideration the other metal (Ery-Hg, and Ery-Pb, respectively), BMI, and blood pressure; the OR for one μg/L increase in Ery-Pb was 1.012 (95% CI 1.001-1.022), and for Ery-Hg 0.90 (95% CI 0.76-1.06). The corresponding result for Ery-Cd when taking into consideration the other two metals, BMI, blood pressure, and smoking, was 1.05 (95% CI 0.69-1.60). The effect modification by time between sampling and end-stage renal disease diagnosis on the associations between Cd, Pb, and Hg concentrations and end-stage renal disease was further investigated within subsets of time between sampling and end-stage renal disease defined by quartile limits (See Additional file 1: Figure S3).

## Discussion

This is the first population-based study with exposure biomarkers and a prospective design assessing the potential associations between risk of developing end-stage renal disease and low-level exposure to the metals Cd, Pb, and Hg. It indicates that the Pb concentration in blood (erythrocytes) is a predictor of development of end-stage renal disease in the general population. Causality is, however, not demonstrated, though one possibility is that Pb decreases the kidney’s ability to resist other factors. In addition, Ery-Cd tended to be related to an increased risk of end-stage renal disease, but confounding by Pb and Hg could partly explain this. In contrast, Ery-Hg showed a negative association. In addition, gender-specific analyses showed that men carried almost all of the Ery-Pb and Ery-Cd associated risks.

One strength of this study is the prospective design, with population-based blood samples collected several years before diagnosis of end-stage renal disease. In addition, end-stage renal disease is a well-defined, hard renal end-point, and the Swedish Renal Registry has been validated, showing that > 95% of the patients who started active end-stage renal disease treatment were reported to the SRR. Thirdly, as is shown by results for reference materials, the quality of our metal analyses was very good.

One weakness of this study is that neither renal function (e.g., estimated glomerular filtration rate) nor urinary albumin were available at baseline. Thus, risk estimates were not adjusted for any impairment of kidney function already existing before the sampling. At normal kidney function glomerular filtration rate is above 90 ml/min/1.73 m^2^. Renal replacement therapy is generally begun at 5–10 ml/min/1.73 m^2^. The rate of decline in renal function in a general healthy population older than 30 years is approximately 0.8 ml/min/1.73 m^2^/year [31]; meanwhile, patients with pre-existing mild to moderate kidney disease (chronic kidney disease 3–4) may lose 2 to 13 ml/min/1.73 m^2^/year [32]. Thus, several of the cases probably had reduced renal function already at the time of sampling.

Another weakness is the relatively few cases of end-stage renal disease, which limits the ability to determine whether risk estimates are homogeneous between the cohorts. From a risk-assessment perspective, an additional third weakness is that the Ery-Pb has decreased over time [33]. Therefore, the measured erythrocyte concentrations neither reflect a lifetime mean, nor the level at time of development of end-stage renal disease. Participation rates in the cohorts were between 45-77%. It is unlikely that any over- or under- representation would affect the risk estimates.

The referents were drawn from the general population, thus having normal values for Pb, Cd, and Hg. In an international perspective, the loads of Pb and Cd in Sweden have been low, while the load of Hg is relatively high [11,34]. The levels of Pb and Cd in erythrocytes are usually about twice as high as those found in whole blood (variation in erythrocyte volume fraction, of course, affect this, but only to a negligible extent), while for Hg, the measured concentrations are closer to those in blood, but depend on the rates between methylmercury and inorganic mercury. For comparability with studies using urinary Cd, it can be noted that 50–60 year-old women from northern Sweden had a median Cd concentration in urine of 0.189 (range 0.03-0.84) μg/L, and in whole blood 0.24 (range: 0.08-2.59) μg/L (corresponding to ca 0.49 μg/L in erythrocytes) [35].

Associations between Cd and Pb and end-stage renal disease have been found in earlier studies using less adequate methods. One previous Swedish study combining occupational and ecological exposure assessment indicated increased risk of developing end-stage renal disease after Cd exposure [15], while another ecological study in Japan did not show any association between mortality from renal failure and Cd levels in local brown rice [36]. These studies are weakened by their lack of individual biomarker data. Cross-sectional studies have found higher levels of Ery-Cd and Ery-Pb in hemodialysis patients than in healthy referents (Taiwan: [17]; U.S.: [18]; Pakistan: [19]; China: [20]). In addition, progression of renal failure was associated with blood-Pb concentration (China: [21,22]) and a slower decline in renal function have been observed after lead chelation therapy (Taiwan: [37]). In most of these countries, the Pb exposure has been higher than in Sweden. On the other hand, in another Swedish study, there was no association between a history of occupational Pb exposure, as assessed by a job-exposure matrix, and risk of end-stage renal disease [38].

For less-severe kidney effects, several studies indicate associations with biomarkers of Cd [8] and Pb [11] exposure. A cross-sectional study in Taiwan showed significant associations between blood-Pb and risk of renal dysfunction and hyperuricemia [39]. Likewise, studies of U.S. adults displayed associations between Cd and Pb in blood on the one hand, and both albuminuria and reduced estimated glomerular filtration rate, on the other [40], for blood-Pb also in adolescents [14].

The evidence for the development of nephropathy at high and prolonged Pb exposure was described as ‘anecdotic’ in a relatively recent review, while data on the relationship between Pb and end-stage renal disease was considered scarce [41]. However, some of the most recent cross-sectional studies were not included in the review. Thus, taking all the recent studies and our present results into account, such an association is likely, even at fairly low-level exposure.

Information on Hg is scarce, but not in conflict with our data, although subjects with occupational exposure to elemental Hg vapor have been shown to display albuminuria [9]. Also, high exposure to inorganic Hg may cause severe membranous nephropathy [23]. However, such high-level exposure to Hg is rare in Sweden. In the general population, the major exposure is to elemental Hg from dental amalgam and methyl-Hg through fish consumption, in both cases at relatively low exposures, not expected to be sufficient to cause renal effects.

It has been claimed that Cd and Pb may cause a general worsening of renal disease [42]. Our results tend to support this, since the patterns were similar for Ery-Pb and Ery-Cd in several primary causes of end-stage renal disease. However, an exception was cases with diabetic nephropathy. Despite the fact that previous studies suggest that Cd may induce diabetes [43,44], and aggravate diabetic renal complications [12], we did not find a difference in Ery-Cd between cases with diabetic nephropathy and referents (only 19 cases).

The results contain two unexpected findings: first a gender difference for all three metals, and second, a negative association between Ery-Hg and the risk of developing end-stage renal disease. The difference in risk estimates for men and women could be due to a gender difference in susceptibility or a difference in exposure biomarkers reliability between men and women. This should be further evaluated. The possibility of confounding by unobserved covariates should be considered for both Ery-Pb and Ery-Hg. A truly protective effect of Hg appears unlikely. Instead, it can be speculated that there may be confounding by a healthy lifestyle, including fish consumption and with a low burden of other risk factors [45] such as low socio-economic status [38,46,47]. An alternative, and even more speculative interpretation, is a protective effect of long-chain omega-3 polyunsaturated fatty acids (PUFAs) from fish. PUFAs correlate to Ery-Hg [48] and have been suggested in treatment of kidney disease, i.e., IgA-nefritis [49,50].

If confounding by healthy life-style is a possible explanation of the results for Hg, such confounding must be considered also for Pb. Ery-Hg and Ery-Pb were positively correlated, and it has previously been observed that Ery-Pb is associated with fish consumption ([33]; possibly due to co-variation with leafy vegetables, a source of dietary Pb). Therefore, the association between end-stage renal disease and Ery-Pb is not likely to be due to confounding by a healthy life-style, since the risk estimate for Ery-Pb then would rather decrease than increase. Significant confounding by occupational factors is also unlikely because occupational exposure could only explain a minor fraction of the cases in this population.

As previously mentioned, several of the cases probably had reduced renal function already at baseline. We therefore performed subgroup analyses within quartile limits of time between sampling and end-stage renal disease (see Additional file 1). The impact of time from sampling to diagnosis only had a limited effect on the odds ratio for Ery-Pb, but for Ery-Cd the odds ratio was lower among individuals with a long time period from sampling to diagnosis. No consistent pattern was seen for Ery-Hg. These results do not indicate that the results for Ery-Pb and Ery-Hg reflect reverse causation, while this may be the case for Ery-Cd. A previously published study showing an association between bone-Pb, which has a very slow turnover, and end-stage renal disease [18] is also in accordance with our finding on Pb.

Both the present and other studies show an increased risk of developing end-stage renal disease in smokers [38,46,47]. Smoking is, however, also an important source of Cd. When both smoking and Cd were included in a multiple analysis, both were statistically significant, thus the possibility remains that other smoking-related factors than Cd may cause the association. The number of never-smokers in the present study is too small to allow a separate analysis of these individuals.

Although it is still uncertain if Pb is truly a cause of end-stage renal disease, this first low-level study using prospectively collected samples for exposure assessment shows associations that give reasons for concern.

## Conclusions

Lead in erythrocytes and blood is associated with end-stage renal disease, even at low levels, as is shown both in this prospective population-based study and in previous cross-sectional studies, and supported by studies of chronic kidney disease. Pb exposure may therefore increase the risk of developing end-stage renal disease in the general population, but further studies are needed to evaluate causality. The case might be similar for Cd, but we found only slight indications for that. Gender-specific analyses indicate potential differences in susceptibility or in exposure biomarker reliability.

## Abbreviations

Cd: Cadmium; Pb: Lead; Hg: Mercury; Ery-Cd: Erythrocyte cadmium concentration; Ery-Pb: Erythrocyte lead concentration; Ery-Hg: Erythrocyte mercury concentration; MONICA: Northern Sweden WHO monitoring of trends and cardiovascular disease; SRR: Swedish renal registry; ICP-MS: Inductively coupled plasma-mass spectrometry; OR: Odds ratio; CI: Confidence interval.

## Competing interests

The authors declare that they have no competing interests.

## Authors’ contributions

JNS carried out statistical analyses, drafted the first manuscript version, and coordinated the manuscript preparation together with BB and IAB. MS took part in manuscript preparation and participated in the design of the study. BB took part in data management. SE, GH, and S Schön took part in study design and data collection. TL carried out all laboratory analyses. S Skerfving and IAB took part in study design and manuscript preparation. All authors contributed to the interpretation of the results, read, and approved the final manuscript.

## Supplementary Material

Additional file 1 End-stage renal disease and low level exposure to lead, cadmium and mercury; a population-based, prospective nested case-referent study in Sweden. Click here for file
