# Supplementary material for: End-stage renal disease and low level exposure to lead, cadmium and mercury; a population-based, prospective nested case-referent study in Sweden
Source: Environ Health. 2013 Jan 23;12:9. doi: 10.1186/1476-069X-12-9 (PMC3566948; doi:10.1186/1476-069X-12-9)
Supplement: Additional file 1 — End-stage renal disease and low level exposure to lead, cadmium and mercury; a population-based, prospective nested case-referent study in Sweden. [file 1476-069X-12-9-S1.doc]

# **Supplemental material**

**End-stage renal disease and low level exposure to lead, cadmium and mercury; a population-based, prospective nested case-referent study in Sweden**

**Johan Nilsson Sommar, Maria Svensson, Bodil Björ, Sölve Elmståhl, Göran Hallmans, Thomas Lundh, Staffan Schön, Staffan Skerfving, Ingvar A Bergdahl**

**Table 1. Metal concentrations in erythrocytes in cases that later in life developed end-stage renal disease and their referents, divided into primary causes of end-stage renal disease**

| **Primary cause of ESRD** |  | **Ery-Cd (µg/L) geometric mean (geometric SD)** |  |  | **Ery-Pb (µg/L) geometric mean (geometric SD)** |  |  | **Ery-Hg (µg/L) mean (SD)** |  |
| --- | --- | --- | --- | --- | --- | --- | --- | --- | --- |
|  | Number of cases | Cases | Referents | Number of cases | Cases | Referents | Number of cases | Cases | Referents |
| All | 118 | 0.86 (2.39) | 0.66 (2.40) | 118 | 66.2 (1.68) | 55.0 (1.67) | 86 | 2.44 (1.64) | 3.07 (2.65) |
| Glomerulonephritis | 32 | 0.93 (2.63) | 0.56 (2.52) | 32 | 65.1 (1.79) | 55.7 (1.65) | 25 | 2.34 (1.49) | 3.10 (2.65) |
| Diabetes nephropathy | 19 | 0.84 (2.24) | 0.66 (2.29) | 19 | 58.9 (1.40) | 60.8 (1.60) | 12 | 2.61 (2.32) | 3.22 (2.31) |
| Nephrosclerosis | 12 | 1.04 (2.11) | 0.73 (2.10) | 12 | 79.0 (1.40) | 56.1 (1.70) | 10 | 2.10 (1.04) | 3.09 (3.02) |
| Pyelonephritis | 5 | 0.95 (2.11) | 0.63 (1.75) | 5 | 74.6 (1.81) | 55.3 (1.78) | 4 | 1.90 (0.97) | 3.10 (1.9) |
| Hereditary kidney disease | 13 | 1.02 (2.72) | 0.74 (2.57) | 13 | 79.2 (1.27) | 58.2 (1.77) | 10 | 3.18 (1.88) | 3.52 (2.66) |
| Other specified | 19 | 0.75 (2.28) | 0.74 (2.31) | 19 | 59.7 (1.64) | 52.4 (1.68) | 10 | 1.95 (1.24) | 3.18 (3.96) |
| Non-specified | 18 | 0.68 (2.10) | 0.80 (2.54) | 18 | 64.9 (2.00) | 54.3 (1.67) | 15 | 2.66 (1.84) | 2.54 (1.78) |

SD=standard deviation

Table 2. Associations (Spearman´s rank correlation coefficients or odds ratios (OR) per unit increase) between data recorded at the baseline examination of cases and referents. Estimates are presented with 95% confidence intervals.

|  | Ery-Cd (µg/L) | Ery-Pb (µg/L) | Ery-Hg (µg/L) | BMI (kg/m2) | Smoking | Age (years) | Diabetes (y/n) |
| --- | --- | --- | --- | --- | --- | --- | --- |
| Ery-Pb (µg/L) | 0.17 (0.09, 0.26) |  |  |  |  |  |  |
| Ery-Hg (µg/L) | -0.03 (-0.14, 0.08) | 0.09 (-0.01, 0.20) |  |  |  |  |  |
| BMI (kg/m^2^) | -0.08 (-0.17, 0.01) | -0.05 (-0.14, 0.04) | -0.09 (-0.20, 0.02) |  |  |  |  |
| Smoking | 0.32 (0.23, 0.40) | 0.10 (0.01, 0.19) | 0.00 (-0.11, 0.11) | 0.00 (-0.10, 0.09) |  |  |  |
| Age (years) | 0.11 (0.02, 0.20) | 0.12 (0.03, 0.21) | 0.01 (-0.10, 0.11) | 0.15 (0.06, 0.24) | 0.12 (0.03, 0.22) |  |  |
| Diabetes (y/n) | 1.38 (0.89, 2.15) | 1.00 (0.98, 1.01) | 0.93 (0.69, 1.25) | 1.12 (1.00, 1.25) | 4.73 (1.37, 16.3) | 1.17 (0.86, 1.61) |  |
| Hypertension^a^ (y/n) | 0.96 (0.79, 1.16) | 1.00 (1.00, 1.01) | 0.97 (0.86, 1.10) | 1.00 (0.94, 1.05) | 1.53 (0.78, 3.01) | 1.40 (0.75, 2.59) | 1.97 (0.85, 4.66) |

Ery-Cd=erythrocyte cadmium concentration; Ery-Pb= erythrocyte lead concentration; Ery-Hg= erythrocyte mercury concentration; BMI=Body Mass Index. OR=odds ratio for a unit increase. Smoking consists of four categories where ORs are given for current- compared to never-smokers. For the association between hypertension and diabetes, the OR for hypertension was given for diabetics compared with non-diabetics.

^a^Hypertension was defined as SBP >140 or DBP > 90 mm Hg, or antihypertensive medication at baseline.

**Figure 1. Conditional logistic regression using penalized cubic splines as well as quartile categorization was used to investigate linearity between log-odds and exposure variables. Log-odds curves are for 1 µg/L increase.**

**Figure 2a**

**Figure 2b**

**Figure 2c**

**Figure 2. Effect modification for each metal exposure variable by the two other respective metals. Odds ratios are for 1 µg/L increase of Ery-Cd (Figure 1a), Ery-Pb (Figure 1b) and Ery-Hg (Figure 1c).**

**Figure 3a**

**Figure 3b**

**Figure 3c**

**Figure 3. Effect modification of ESRD by time between sampling and diagnosis, odds ratio (OR) for 1 µg/L increase of Ery-Cd (Figure 3a), Ery-Pb (Figure 3b) and Ery-Hg (Figure 3c). The estimates are ORs, first for all data, and then for subsets of cases with time between sampling and ESRD above quartile limits. The ORs for Ery-Pb and Ery-Hg are adjusted for the other metal (i.e. Ery-Hg and Ery-Pb, respectively), BMI and blood pressure, and Ery-Cd was adjusted for the other two metals, BMI, blood pressure and also smoking. Due to limited number of cases within each subset, diabetes was not included as covariate in these analyses.**
